# Influencing factors and path analysis of posttraumatic growth in patients with autoimmune diseases

**DOI:** 10.3389/fpsyt.2026.1830401

**Published:** 2026-06-10

**Authors:** Zheyuan Xia, Leran Tang, Ting Yao, Qiao Hu, Xiao Wang, Xiang Wang

**Affiliations:** 1 The School of Nursing, Anhui University of Chinese Medicine, Hefei, China; 2 Laboratory of Geriatric Nursing and Health, Anhui University of Chinese Medicine, Hefei, China

**Keywords:** autoimmune diseases, fear of disease progression, nursing care, path analysis, posttraumatic growth

## Abstract

**Objective:**

Guided by the Transactional Model of Stress and Coping, this study aimed to construct a structural equation model of posttraumatic growth in patients with autoimmune diseases. Through path analysis, the associative pathways among key factors, including fear of disease progression, social support, illness perception, and coping modes, were examined.

**Methods:**

A cross-sectional study was conducted. A total of 317 patients with autoimmune diseases were recruited from the rheumatology and immunology departments of three tertiary grade A hospitals in Anhui Province, China, between January 2025 and February 2026 using a convenience sampling method. Participants were assessed using a general information questionnaire, the Posttraumatic Growth Inventory (PTGI), the Fear of Progression Questionnaire (FoP-Q), the Perceived Social Support Scale (PSSS), the Medical Coping Modes Questionnaire (MCMQ), the Brief Illness Perception Questionnaire (BIPQ), and a Visual Analog Scale (VAS) for disease activity. Data were analyzed using SPSS 26.0 for correlation and regression analyses. Structural equation modeling was performed using Amos 26.0, and the bias-corrected bootstrap method was employed to test mediating effects.

**Results:**

A total of 288 patients completed the survey. The total score of posttraumatic growth was 52.20 ± 13.87. The structural equation model demonstrated a good fit to the data (χ²/df = 2.736, CFI = 0.935, TLI = 0.928, RMSEA = 0.063) and accounted for 49.3% of the variance in posttraumatic growth. Fear of disease progression was directly positively associated with posttraumatic growth (β = 0.290, 95% CI: 0.191–0.392), accounting for 68.7% of its total effect. Mediation analysis revealed three statistically significant mediating pathways: (1) fear of disease progression → social support → posttraumatic growth (indirect effect = 0.054, 95% CI: 0.015–0.107, P = 0.006), accounting for 12.80% of the total effect; (2) fear of disease progression → patient-reported disease activity → posttraumatic growth (indirect effect = 0.048, 95% CI: 0.015–0.099, P = 0.003), accounting for 11.37%; and (3) fear of disease progression → avoidance coping → posttraumatic growth (indirect effect = 0.037, 95% CI: 0.011–0.080, P = 0.004), accounting for 8.77%. The mediating effects of fear of disease progression via resignation coping and illness perception were not statistically significant (P > 0.05). These findings suggest that fear of disease progression is positively associated with posttraumatic growth and may be indirectly associated through a facilitative pathway via social support as well as inhibitory pathways via patient-reported disease activity and avoidance coping.

**Conclusion:**

Fear of disease progression is positively associated with posttraumatic growth in patients with autoimmune diseases. In clinical practice, attending to patients’ level of fear of disease progression, strengthening social support systems, guiding reduction of avoidance coping, and alleviating negative illness perception are all associated with promoting patients’ posttraumatic growth and positive psychological adaptation. Given the cross-sectional design of this study, the above associations should be understood as covarying relationships among variables rather than causal effects.

## 1 Introduction

Autoimmune diseases (ADs) are a group of chronic, systemic inflammatory conditions characterized by the immune system aberrantly attacking the body’s own tissues. These diseases encompass various types, including systemic lupus erythematosus, rheumatoid arthritis, systemic sclerosis, and ankylosing spondylitis (1). Typically, these diseases are marked by a prolonged course, recurrent episodes, and are accompanied by chronic pain, physical functional limitations, and potential multi-organ damage, imposing a persistent physical and psychological burden on patients (2). As a significant negative life event, the diagnosis and long-term management of an autoimmune disease itself constitutes a continuous psychological stressor, predisposing patients to psychological distress such as anxiety, depression, and fear (3, 4).

Notably, disease-related trauma does not inevitably lead to negative psychological outcomes. During the long-term process of adaptation, some patients with ADs experience positive psychological changes in dimensions such as self-perception, interpersonal relationships, and meaning of life, a phenomenon defined as posttraumatic growth (PTG) (5). PTG not only alleviates disease-related psychological distress but also enhances treatment adherence and quality of life, serving as a key psychological marker of positive disease adaptation (6). With advances in therapeutic approaches, patients with ADs have entered a new phase of “long-term survival with the disease.” Therefore, in-depth investigation into the factors associated with PTG in this population is of great significance for promoting long-term psychological adaptation and holistic rehabilitation.

The Transactional Model of Stress and Coping provides a robust framework for understanding the pathways associated with PTG. This model posits that the psychological outcomes experienced by individuals confronting stressors are not a direct consequence of the stressors themselves, but rather the result of a complex interplay and dynamic modulation among internal and external factors, including cognitive appraisal, social support, and coping strategies (7). For patients with ADs, fear of disease progression represents a core psychological stressor (8). As a “motivational” emotion, this fear possesses a dual nature: at moderate levels, it can enhance patients’ vigilance regarding their illness, prompting them to adopt proactive coping behaviors to mitigate the risk of deterioration. Conversely, excessive fear can steer patients toward avoidance coping, thereby inhibiting psychological growth. This dual attribute renders its pathways of influence on PTG particularly complex, necessitating systematic investigation. According to the Transactional Model of Stress and Coping, psychological outcomes of stress are co-regulated by three core factors: cognitive appraisal, social support, and coping strategies. Previous empirical studies have also identified social support, coping modes, and illness perception as key mediating variables through which fear may exert its effects. Social support, as a crucial external protective factor, can buffer the inhibitory effect of negative emotions on psychological growth by providing emotional and tangible assistance (9). Medical coping modes, serving as intrinsic cognitive and behavioral regulation strategies, play a significant moderating role in stress responses, with different patterns (e.g., confrontation, avoidance) exerting distinct influences (10). Illness perception, as the core component of cognitive appraisal, acts as a pivotal mediating variable in the pathway connecting stressors to psychological adaptation outcomes (11).

However, to the best of our knowledge, no previous study has simultaneously examined both the facilitative and inhibitory pathways associated with PTG among patients with autoimmune diseases within a single structural equation modeling framework. Furthermore, existing study samples have been largely confined to a single disease type, and the limited sample sizes have restricted the construction and generalizability of the models.

Systemic lupus erythematosus, systemic sclerosis, and rheumatoid arthritis are representative conditions within the AD spectrum characterized by both high prevalence and complex management requirements. Despite differences in disease pathology, patients share substantial commonalities across stressor dimensions, including illness uncertainty, altered body image, and persistent concerns about disease progression, making them an ideal sample for exploring transdiagnostic psychological factors related to PTG. Therefore, guided by the Transactional Model of Stress and Coping, this study focused on patients with these three conditions to construct and validate an integrated model. We systematically examined both the facilitative and inhibitory pathways through which fear of disease progression, social support, medical coping modes, and illness perception are associated with PTG, aiming to delineate the direction and nature of these associations and provide an empirical basis for developing targeted clinical psychological interventions.

## 2 Subjects and methods

### 2.1 Study design and participants

Between January 2025 and February 2026, patients with autoimmune diseases were recruited from the rheumatology and immunology departments of three tertiary grade A hospitals in Anhui Province, China, using a convenience sampling method. A total of 365 eligible patients were invited, of whom 48 declined to participate due to time conflicts, lack of interest, or acute disease exacerbation. Finally, 317 patients agreed to participate and signed informed consent forms, yielding an agreement rate of 86.8% (317/365). Among these 317 participants, 288 completed the questionnaires, with a completion rate of 90.8%.

Inclusion criteria: (1) a confirmed diagnosis of one of the following ADs: systemic lupus erythematosus, systemic sclerosis, or rheumatoid arthritis. The specific diagnostic criteria were as follows: for SLE, the 2019 European League Against Rheumatism/American College of Rheumatology (EULAR/ACR) classification criteria (12); for SSc, the 2013 American College of Rheumatology/European League Against Rheumatism (ACR/EULAR) classification criteria (13); and for RA, the 2010 American College of Rheumatology/European League Against Rheumatism (ACR/EULAR) classification criteria (14). (2) age ≥ 18 years; (3) disease duration ≥ 1 year (ensuring that patients had experienced long-term trauma associated with their illness); (4) intact consciousness and basic abilities to read, comprehend, and communicate; and (5) provision of informed consent and voluntary participation in the study.

Exclusion criteria: (1) presence of other severe autoimmune diseases, malignant tumors, or serious cardiovascular or cerebrovascular diseases; or being in the acute phase of the disease. The acute phase was defined as the presence of active organ involvement as determined by a rheumatologist (e.g., active lupus nephritis, acute exacerbation of interstitial pneumonia in systemic sclerosis, severe joint swelling in rheumatoid arthritis accompanied by elevated acute-phase reactants such as C-reactive protein), or hospitalization due to the above active conditions. (2) severe dependence in activities of daily living, as assessed by the Barthel Index (15) (Barthel Index score ≤ 40); (3) experience of a traumatic event within the past year, such as severe natural disasters, accidents, or the death of a family member; (4) previous or current receipt of psychological intervention.

Sample size estimation: Based on the sample size requirements for structural equation modeling, the recommended sample size is typically at least 10 times the number of observed variables or 10–20 times the number of free parameters (16). The proposed model in this study includes 19 observed variables; therefore, the required sample size was estimated to range from 190 to 380. Considering the expected effective questionnaire recovery rate (≥90%), the target sample size was planned to be between 209 and 418 participants.

### 2.2 Study procedure

The research team consisted of six members, with responsibilities spanning study management, quality control, data collection, and statistical analysis. All team members had prior experience in community-based epidemiological surveys and received standardized training prior to the study. The training covered questionnaire interpretation, communication skills, and data entry protocols; only those who passed a simulated survey assessment were authorized to participate in the formal investigation.

All participants provided written informed consent. For individuals unable to read independently, the consent form was read aloud by the investigator, and a fingerprint was used in lieu of a signature in the presence of an independent witness. Surveys were administered in quiet hospital wards or in participants’ home environments using a one-on-one format, with each session lasting 15–25 minutes. The majority of participants completed the questionnaires independently; for those who experienced difficulties, investigators provided assistance in a neutral manner.

Questionnaires were reviewed and entered into the database within 24 hours of collection, with any ambiguous responses verified promptly. Questionnaires were excluded if the completion rate was <90%, the completion time was <10 minutes, or if responses exhibited a patterned regularity. Data entry was performed independently by two researchers to ensure accuracy.

### 2.3 Variables and instruments

This study was guided by the Transactional Model of Stress and Coping, and the selection of influencing factors was informed by a systematic literature review. After discussion within the research team, the following variables were ultimately included in the study model: fear of disease progression, social support, medical coping modes, illness perception, and patient global assessment of disease activity. Sociodemographic and clinical characteristics were collected using a general information questionnaire. Variables including PTG, fear of disease progression, social support, illness perception, and medical coping modes were assessed using standardized scales with established reliability and validity in both Chinese and international contexts. Patient global assessment of disease activity was measured using a Visual Analog Scale (VAS).

#### 2.3.1 Demographic and clinical instrument

Based on a literature review, a general information questionnaire was designed to collect sociodemographic and clinical data. The sociodemographic variables included six items: gender, age, educational level, employment status, marital status, and fertility status. The disease-related variables included seven items: type of autoimmune disease, disease duration (years), number of comorbidities, number of medications taken, financial burden imposed by the disease, ability to perform activities of daily living, and hospitalization history within the past year.

#### 2.3.2 Simplified Chinese version of the posttraumatic growth inventory

The scale was originally developed by Tedeschi in 1996 (5) to assess the positive psychological changes experienced by individuals following traumatic events. The Chinese version of the scale has been applied and validated in populations such as adults with congenital heart disease (17, 18). The scale comprises 20 items across five dimensions: Relating to Others (6 items), New Possibilities (3 items), Personal Strength (4 items), Spiritual Change (3 items), and Appreciation of Life (4 items). Items are rated on a 6-point Likert scale ranging from 0 (“I did not experience this change as a result of my crisis”) to 5 (“I experienced this change to a very great degree as a result of my crisis”), yielding a total score between 0 and 100. Higher scores indicate greater levels of PTG. In this study, the Cronbach’s α coefficient for the scale was 0.85.

#### 2.3.3 Fear of progression questionnaire

The scale was originally developed by Mehnert et al. in 2006 (19) to assess an individual’s fear regarding disease progression, recurrence, and the consequent physical, psychological, and social functional impairments. The Chinese version of the scale has been applied and validated in populations such as systemic lupus erythematosus (20). The scale comprises 12 items across two dimensions: Fear about Physical Health (7 items) and Fear about Social and Family Life (5 items). Items are rated on a 5-point Likert scale ranging from 1 (“never”) to 5 (“very often”), yielding a total score between 12 and 60. Higher scores indicate greater fear of disease progression. In this study, the Cronbach’s α coefficient for the scale was 0.873.

#### 2.3.4 Perceived social support scale

The scale was developed by Zimet et al. (21) to measure the extent to which an individual perceives support from family, friends, and significant others. The Chinese version of the scale has been applied and validated in populations such as rheumatoid arthritis (22). The scale comprises 12 items across three dimensions: Family Support (4 items), Friend Support (4 items), and Significant Other Support (4 items). Items are rated on a 7-point Likert scale ranging from 1 (“very strongly disagree”) to 7 (“very strongly agree”), yielding a total score between 12 and 84. Higher scores indicate greater levels of perceived social support. Scores of 12–36 are classified as low support, 37–60 as moderate support, and 61–84 as high support. In this study, the Cronbach’s α coefficient for the scale was 0.896.

#### 2.3.5 Brief illness perception questionnaire

The scale was developed by Broadbent et al. (23) to rapidly assess patients’ subjective cognitive and emotional representations of their illness. The Chinese version of the scale has been applied and validated in populations such as rheumatoid arthritis (24). The scale comprises 8 items across three dimensions: Cognitive Representations (5 items), Emotional Representations (2 items), and Illness Comprehensibility (1 item). Items are rated on an 11-point Likert scale ranging from 0 (e.g., “not at all”/”no effect at all”) to 10 (e.g., “extremely”/”severely”), yielding a total score between 0 and 80. Higher scores indicate more negative illness perceptions and greater perceived symptom burden. In this study, the Cronbach’s α coefficient for the scale was 0.79.

#### 2.3.6 Medical coping modes questionnaire

The scale was developed by Feifel et al. (25) to assess the coping strategies individuals adopt when facing illness. The Chinese version of the scale has been applied and validated in populations such as systemic lupus erythematosus (26). The scale comprises 20 items across three dimensions: Confrontation (8 items), Avoidance (7 items), and Resignation (5 items). Items are rated on a 4-point Likert scale ranging from 1 (“never”) to 4 (“always”), yielding a total score between 20 and 80. The dimension with the highest score indicates the predominant coping mode the individual employs in response to their current illness condition. In this study, the Cronbach’s α coefficient for the scale was 0.81.

#### 2.3.7 Patient global assessment of disease activity, PtGA

Patient global assessment disease activity was measured using a Visual Analog Scale (VAS) (27). Patients were instructed to mark the point on a 10 cm line that best corresponded to their overall perception of disease activity over the past week. The line was anchored with “0” (no activity) at one end and “10” (highly active) at the other. The distance from the zero endpoint to the patient’s mark was measured and recorded to one decimal place, yielding a score ranging from 0 to 10. Higher scores indicated greater self-perceived disease activity.

### 2.4 Statistical analysis

Data were double-checked and entered by two researchers independently. Statistical analyses were performed using SPSS version 26.0. Harman’s single-factor test was conducted to assess common method bias. Univariate analysis was performed using independent samples t-tests or one-way analysis of variance (ANOVA), as appropriate. Pearson correlation analysis was used to examine bivariate correlations among variables. Multivariate analysis was conducted using multiple linear regression.

Structural equation modeling (SEM) was performed using AMOS version 26.0. Model fit was evaluated using the following indices: chi-square to degrees of freedom ratio (χ²/df), comparative fit index (CFI), Tucker–Lewis index (TLI), and root mean square error of approximation (RMSEA). The criteria for acceptable model fit were: χ²/df < 3.00, CFI > 0.90, TLI > 0.90, and RMSEA < 0.08.

The significance of mediating effects was tested using the bias-corrected bootstrap method with 5,000 resamples. Indirect effects were considered statistically significant if the 95% confidence interval (CI) did not include zero. A two-tailed P-value < 0.05 was considered statistically significant for all analyses.

### 2.5 Theoretical model construction

**Figure 1:**
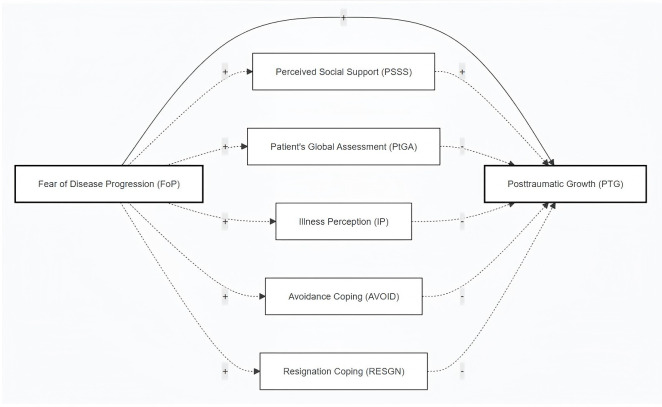
Hypothesized theoretical model of posttraumatic growth in patients with autoimmune diseases. FoP, Fear of Disease Progression; PSSS, Perceived Social Support; PtGA, Patient's Global Assessment of Disease Activity; IP, Illness Perception; AVOID, Avoidance Coping; RESGN, Resignation Coping; PTG, Posttraumatic Growth. Solid arrow indicates hypothesized direct effect; dashed arrows indicate hypothesized indirect (mediating) effects. “+” indicates a hypothesized positive relationship; “−” a hypothesized negative relationship.

Based on the Transactional Model of Stress and Coping, combined with a systematic literature review and theoretical associations among variables, this study constructed a theoretical hypothesis model consisting of one independent variable, five mediating variables, and one dependent variable (**Figure 1**). In this model, fear of disease progression (FoP) serves as the core independent variable, and posttraumatic growth (PTG) as the outcome variable. Perceived social support (PSSS), patient’s global assessment of disease activity (PtGA), avoidance coping (AVOID), resignation coping (RESGN), and illness perception (IP) are included as mediating variables. The hypothesized pathways are as follows: ① fear of disease progression is directly and positively associated with posttraumatic growth; ② fear of disease progression is associated with posttraumatic growth through the positive mediating role of social support; ③ fear of disease progression is associated with posttraumatic growth through the negative mediating roles of self-assessed disease activity, illness perception, avoidance coping, and resignation coping. Subsequently, structural equation modeling was used to empirically test the above path hypotheses to clarify the direct and indirect associative patterns among the variables.

### 2.6 Ethical considerations

This study was conducted in strict accordance with the principles of the Declaration of Helsinki and was approved by the Ethics Committee of Anhui University of Chinese Medicine (Approval No. AHUCM-HSS-2025008). All participants provided written informed consent after being fully informed about the purpose and procedures of the study. This was an anonymous questionnaire survey, and all collected data were used solely for statistical analysis, with strict protection of patient privacy.

## 3 Results

### 3.1 Demographic and clinical characteristics

A total of 317 questionnaires were distributed, and 288 valid responses were received, yielding an effective response rate of 90.85%. The participants’ ages ranged from 23 to 80 years, with a mean age of 47.89 ± 13.35 years. Among the participants, 227 were patients with RA (170 females, 57 males), accounting for 78.8% of the sample, with a mean age of 50.06 ± 12.68 years; 47 were patients with SLE (44 females, 3 males), comprising 16.3%, with a mean age of 37.76 ± 12.80 years; and 14 were patients with SSc (13 females, 1 male), representing 4.9%, with a mean age of 46.64 ± 10.35 years. Detailed demographic and clinical characteristics are presented in **Table 1**.

**Table 1:** Comparison of PTG scores among ADs patients with different demographic and disease characteristics(n=288).

Variables	Subgroup	N (%)	PTG score (mean ± SD)	F/X^2^	*P* value
Gender	Female	230 (79.9)	52.54±14.09	0.935^1)^	0.351
	Male	58 (20.1)	50.64±12.96		
Age (year)	<45	120 (41.7)	51.33±14.37	0.526^2)^	0.592
	45-59	107 (37.2)	52.29±12.49		
	≥60	61 (21.1)	53.56±15.23		
Education	Senior high school and below	170 (59)	51.21±14.15	-1.394^1)^	0.164
	Bachelor’s degree and above	118 (41)	53.53±13.40		
Employment status	Employed	222 (77.1)	53.14±13.14	2.203^1)^	0.028
	Unemployed/Retired	66 (22.9)	48.88±15.75		
Marital status	Married	230 (79.9)	53.46±13.27	3.210^1)^	0.001
	Single or others	58 (20.1)	47.02±15.08		
Fertility status	Have children	193 (67)	53.72±14.23	2.752^1)^	0.006
	No children	95 (33)	48.99±12.59		
Type of autoimmune disease	RA	227 (78.8)	52.52±14.05	0.817^2)^	0.443
	SLE	47 (16.3)	49.89±13.74		
	SSc	14 (4.9)	53.93±11.08		
Disease Duration (year)^①^	<2	96 (33.3)	51.91±14.76	1.844^2)^	0.160
	2-<5	100 (34.7)	50.46±13.65		
	≥5	92 (32)	54.27±12.99		
Number of comorbidities^②^	0	91 (31.6)	55.16±12.75	5.283^2)^	0.006
	1-2	104 (36.1)	52.64±15.61		
	≥3	93 (32.3)	48.68±12.11		
Number of medications taken^③^	<3	140 (48.6)	53.00±14.30	1.008^1)^	0.314
	≥3	148 (51.4)	51.36±13.45		
Financial burden imposed by the disease	None	51 (17.7)	55.92±15.22	3.762^2)^	0.024
	Mild	109 (37.9)	52.99±14.32		
	High	128 (44.4)	49.95±12.57		
Ability to perform activities of daily living	Independent	200 (69.4)	52.84±13.51	0.951^2)^	0.388
	Mild	69 (24)	51.06±15.76		
	Moderate	19 (6.6)	49.00±9.47		
Hospitalization history within the past year	Yes	78 (27.1)	52.69±15.14	0.397^2)^	0.692
	No	210 (72.9)	51.96±13.40		

^1)^*t* value ^2)^*F* value.

① Disease duration was defined as the time interval from diagnosis to the year of the survey. ② Comorbidities referred to other physical complications clearly diagnosed by clinicians as being attributable to the ADs. ③ Number of medications was defined as the number of distinct drug types regularly taken by the patient for the treatment of the ADs.

### 3.2 Common method bias test

Given that this study employed self-report scales, there was a potential risk of common method bias. To enhance the rigor of the research, Harman’s single-factor test was conducted to examine this issue. The results revealed 21 factors with eigenvalues greater than 1, and the first factor accounted for 19.29% of the total variance, which is below the threshold criterion of 40%. Therefore, no severe common method bias was detected in this study.

### 3.3 Scores of posttraumatic growth, fear of disease progression, perceived social support, illness perception, and coping styles in patients with autoimmune diseases

The total score on the PTGI was 52.20 ± 13.87, with scores ranging from 15 to 85. The mean item scores for each dimension were as follows: Relating to Others, 3.07 ± 0.55; Personal Strength, 2.92 ± 0.59; Appreciation of Life, 2.63 ± 0.37; Spiritual Change, 2.39 ± 0.40; and New Possibilities, 1.95 ± 0.43.

The total score on the FoP-Q-SF was 33.06 ± 8.41, with scores ranging from 14 to 53. The mean item scores for each dimension were as follows: Fear about Physical Health, 2.91 ± 0.63; and Fear about Social and Family Life, 2.72 ± 0.57.

The total score on the PSSS was 44.31 ± 12.65, with scores ranging from 14 to 76. The mean item scores for each dimension were as follows: Family Support, 4.22 ± 1.26; Significant Other Support, 3.67 ± 1.33; and Friend Support, 3.81 ± 1.12.

The total score on the BIPQ was 50.97 ± 13.99, with scores ranging from 20 to 79. The mean item scores for each dimension were as follows: Cognitive Representations, 6.56 ± 1.72; Emotional Representations, 6.40 ± 2.21; and Illness Comprehensibility, 5.22 ± 2.56.

For the MCMQ, the dimension scores were as follows: Confrontation scores ranged from 10 to 26, with a total dimension score of 15.86 ± 3.50 and a mean item score of 1.98 ± 0.44; Avoidance scores ranged from 5 to 19, with a total dimension score of 12.83 ± 3.32 and a mean item score of 1.83 ± 0.47; and Resignation scores ranged from 3 to 19, with a total dimension score of 12.83 ± 3.32 and a mean item score of 2.57 ± 0.66.

The patient self-assessment score for disease activity was 5.91 ± 2.32, with scores ranging from 0 to 10.

### 3.4 Univariate analysis of factors associated with PTG in patients with ADs

Comparisons of PTG scores among patients with ADs revealed statistically significant differences based on employment status, marital status, fertility status, financial burden imposed by the disease, and number of comorbidities (P < 0.05). These results are presented in **Table 1**.

### 3.5 Correlation between PTG and key psychosocial factors in ADs patients

Pearson correlation analysis revealed that PTG in patients with ADs was significantly positively correlated with fear of disease progression and social support, and significantly negatively correlated with disease activity, illness perception, resignation coping, and avoidance coping. No significant correlation was found between PTG and confrontation coping. These results are presented in **Table 2**.

**Table 2:** Correlation analysis between study variables and PTG (r values, N = 288).

Variables	PTG	Fear of disease progression	Social support	Illness perception	Confrontation coping	Avoidance coping	Resignation coping	Disease activity
PTG	1.000							
Fear of disease progression	0.412^2)^	1.000						
Social support	0.396^2)^	0.193^2)^	1.000					
Illness perception	-0.367^2)^	-0.146^1)^	-0.005	1.000				
Confrontation coping	0.103	0.050	0.064	-0.065	1.000			
Avoidance coping	-0.405^2)^	-0.210^2)^	-0.149^1)^	0.166^2)^	-0.037	1.000		
Resignation coping	-0.318^2)^	-0.173^2)^	-0.123^1)^	0.184^2)^	-0.038	0.656^2)^	1.000	
Disease activity	-0.457^2)^	-0.181^2)^	-0.173^2)^	0.36^2)^	-0.093	0.263^2)^	0.193^2)^	1.000

^1)^P<0.05 ^2)^P<0.01.

### 3.6 Multiple linear regression analysis of factors associated with PTG in patients with ADs

A multiple linear regression analysis was conducted with PTG as the dependent variable. Variables that were statistically significant in the univariate and correlation analyses were entered as independent variables (entry criterion α = 0.05, removal criterion α = 0.10). For the multicategorical variables “financial burden” and “number of comorbidities,” dummy variables were created according to the k-1 principle, with “no financial burden” and “0 comorbidities” serving as the reference groups, respectively. For employment status, “unemployed/retired” was used as the reference; for marital status, “single or others” was the reference; and for fertility status, “have children” was the reference. Continuous variables were entered as original values. All analyses were performed using the enter method.

The results revealed that employment status, fertility status, self-assessed disease activity, financial burden, number of comorbidities, fear of disease progression, social support, illness perception, avoidance coping, and resignation coping were all independent influencing factors of PTG in patients with ADs (all P < 0.05). The model collectively explained 49.3% of the total variance in PTG. These results are presented in **Table 3**.

**Table 3:** Multiple linear regression analysis of PTG in Patients with Ads (n=288).

Variable	b	SE	β	t	P	Collinearity statistics	
						Tolerance	Variance inflation factor
Constant	65.478	5.354	–	12.230	<0.001	–	–
Employment status	-3.218	1.398	-0.098	-2.302	0.022	0.981	1.019
Marital status							
Fertility status	-2.919	1.254	-0.099	-2.327	0.021	0.975	1.026
Financial burden imposed by the disease							
Mild	0.363	2.351	0.013	0.154	0.877	0.261	3.835
High	-2.064	2.107	-0.074	-0.980	0.328	0.309	3.233
Number of comorbidities							
1-2	-2.904	2.074	-0.101	-1.400	0.163	0.341	2.929
≥3	-2.631	1.928	-0.089	-1.365	0.173	0.417	2.397
Fear of disease progression	0.385	0.074	0.233	5.222	<0.001	0.885	1.130
Social support	0.288	0.049	0.262	5.811	<0.001	0.869	1.151
Disease activity	-1.236	0.287	-0.207	-4.307	<0.001	0.765	1.308
Illness perception	-0.210	0.046	-0.209	-4.524	<0.001	0.825	1.212
Resignation coping	-0.239	0.200	-0.061	-1.195	0.233	0.673	1.487
Avoidance coping	-0.643	0.195	-0.174	-3.300	0.001	0.635	1.576

R = 0.717, R² = 0.514, Adjusted R² = 0.493, Std. Error of the Estimate = 9.88013, F = 24.222, Durbin-Watson = 2.317, P < 0.001.

Collinearity diagnostics showed that the variance inflation factor (VIF) for all independent variables was less than 5 (ranging from 1.015 to 3.835), and tolerance values were all greater than 0.2, indicating no serious multicollinearity.

### 3.7 Path analysis of factors influencing PTG in patients with ADs

**Figure 2:**
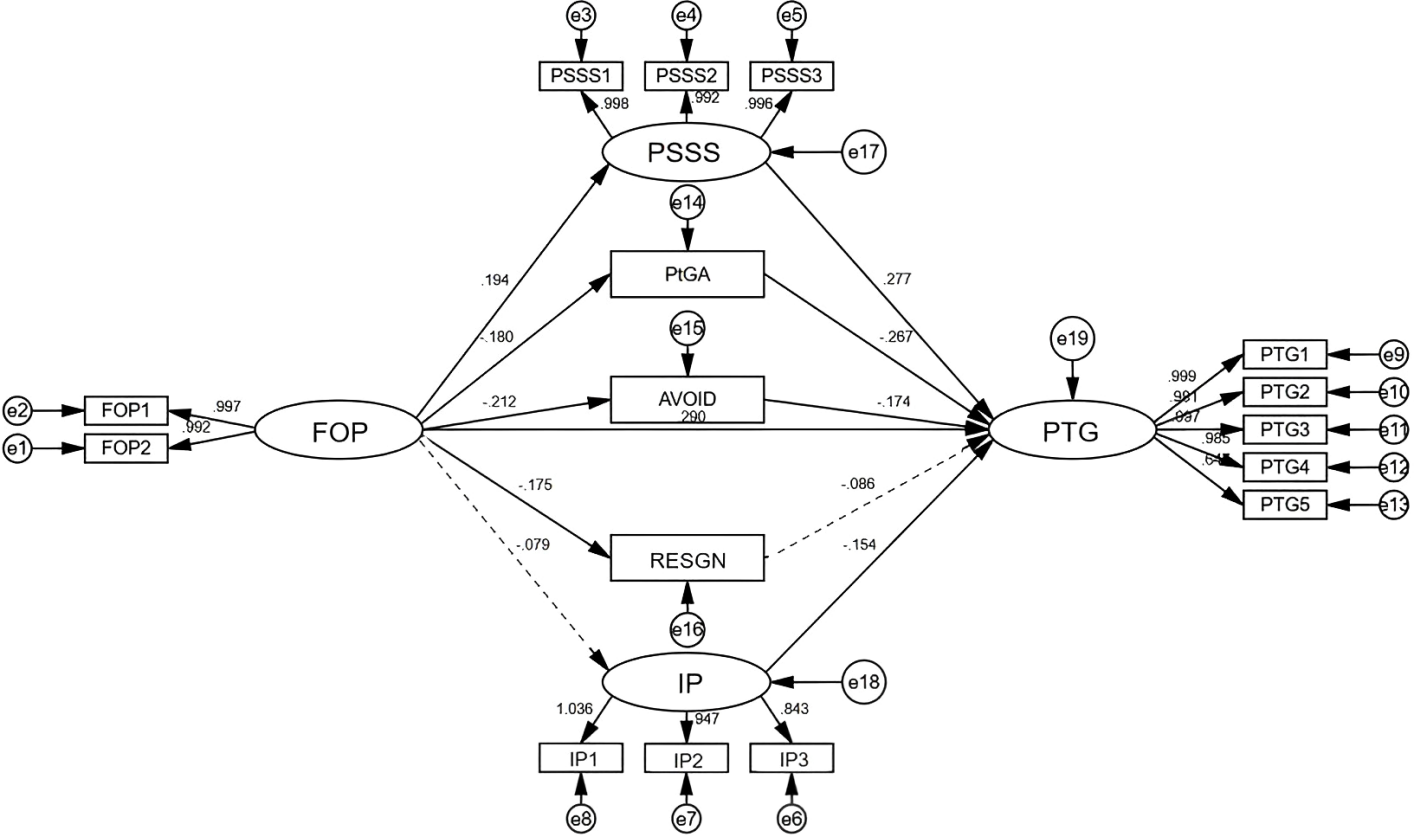
Structural equation model for PTG in ADs patients (standardized). FoP, Fear of Disease Progression; PSSS, Perceived Social Support; PtGA, Patient's Global Assessment of Disease Activity; AVOID, Avoidance Coping; RESGN, Resignation Coping; BIPQ, Brief Illness Perception Questionnaire; PTG, Posttraumatic Growth. Numbers on solid arrows are standardized path coefficients; dashed arrows indicate non-significant paths.

An initial structural equation model was constructed using AMOS version 26.0, with PTG specified as the dependent variable and fear of disease progression (FoP) as the independent variable. Perceived social support scale (PSSS), patient global assessment of disease activity (PtGA), avoidance coping (AVOID), resignation coping (RESGN), and illness perception (IP) were entered as mediating variables. The model was identified, and all variables conformed to a normal distribution. Model fitting was performed using the maximum likelihood method. The fit indices were as follows: χ² = 85.632, χ²/df = 2.123, GFI = 0.956, AGFI = 0.928, RMSEA = 0.060, NFI = 0.943, RFI = 0.925, IFI = 0.968, TLI = 0.956, and CFI = 0.967. The path diagram of the model is presented in **Figure 2**.

The results of the AMOS standardized path analysis revealed that FoP exerted a direct positive effect on PTG in patients with ADs (β = 0.29, P < 0.001). Social support also demonstrated a direct positive effect on PTG (β = 0.31, P < 0.001). In contrast, self-assessed disease activity, avoidance coping, and illness perception each exhibited direct negative effects on PTG (β = -0.22, P < 0.001; β = -0.18, P < 0.01; and β = -0.17, P < 0.01, respectively). The direct effect of resignation coping on PTG was not statistically significant (β = -0.07, P = 0.233).

The mediating effects were tested using the bias-corrected nonparametric percentile bootstrap method with 5,000 resamples. The results revealed that:

① The total effect of FoP on PTG was 0.422 (95% CI: 0.310–0.527). The direct effect was 0.290 (95% CI: 0.191–0.392), accounting for 68.7% of the total effect. The total indirect effect was 0.132 (95% CI: 0.066–0.204), accounting for 31.3% of the total effect.

② The following specific mediating pathways were statistically significant: FoP → Social Support → PTG (indirect effect 1 = 0.054, 95% CI: 0.015–0.107); FoP → Patient Global Assessment of Disease Activity → PTG (indirect effect 2 = 0.048, 95% CI: 0.015–0.099); and FoP → Avoidance Coping → PTG (indirect effect 3 = 0.037, 95% CI: 0.011–0.080).

The mediating effects of the pathways FoP → Resignation Coping → PTG (indirect effect 4 = 0.015, 95% CI: –0.004 to 0.051) and FoP → Illness Perception → PTG (indirect effect 5 = –0.022, 95% CI: –0.052 to 0.004) did not reach statistical significance. These results are presented in **Table 4**.

**Table 4:** Bootstrap test of mediation effect (normalized).

Variable	β	SE	95%CI^1)^	P
Total effect	0.422	0.055	0.310~0.527	<0.001
Direct effect	0.290	0.051	0.191~0.392	<0.001
Total indirect effect	0.132	0.035	0.066~0.204	<0.001
Indirect effect 1 (FOP→PSSS→PTG)	0.054	0.023	0.015~0.107	0.006
Indirect effect 2 (FOP→PtGA→PTG)	0.048	0.020	0.015~0.099	0.003
Indirect effect 3 (FOP→AVOID→PTG)	0.037	0.017	0.011~0.080	0.004
Indirect effect 4 (FOP→RESGN→PTG)	0.015	0.013	-0.004~0.051	0.111
Indirect effect 5 (FOP→IP→PTG)	-0.022	0.014	-0.052~0.004	0.106

1) The bias-corrected nonparametric percentile method was used.

## 4 Discussion

### 4.1 Overall level and factors associated with PTG in patients with ADs

In this study, the mean score for PTG among patients with ADs was 52.20 ± 13.87. This finding is generally consistent with previous reports on patients with SLE (28), RA (29), and Sjögren’s syndrome (30). However, it was lower than the PTG levels reported in a multinational meta-analysis of cancer patients, which yielded pooled mean scores of 61.40 (95% CI: 52.28–70.51) for Asian populations, 59.83 (95% CI: 54.12–65.54) for European populations, and 60.42 (95% CI: 54.68–66.16) for US populations (31).

This discrepancy is related to fundamental differences in disease trajectories and treatment expectations between the two categories of illness, which may correspond to heterogeneity in the pathways through which PTG develops. From the perspective of disease-related stress characteristics, cancer typically follows an acute-linear trajectory of “diagnosis–treatment–remission/recovery.” The presence of a defined treatment endpoint and expectations for recovery is often accompanied by a “survivor” identity, which is in turn associated with positive cognitive restructuring in domains such as appreciation of life and new possibilities. In contrast, ADs are characterized by chronicity, fluctuation, and unpredictable prognosis, constituting a persistent “existential threat.” Patients face no definitive treatment endpoint and remain entrenched in the cumulative effects of illness uncertainty and functional decline over the long term. This diffuse stress burden is associated with a slower integration process of PTG. In other words, while PTG in cancer patients largely reflects the reconstruction of meaning following a “crisis that has been overcome,” patients with ADs must dynamically construct growth amidst “continuously coexisting stress,” confronting more enduring psychological resistance. This distinction suggests caution against the “homogeneity assumption of PTG formation mechanisms” when drawing on psychological intervention experiences from oncology populations. For patients with ADs, interventions may focus on helping them establish anchor points for gradual growth within ongoing uncertainty, fostering a dynamic growth model compatible with living alongside the disease, rather than simply applying the linear “post-crisis rebirth” framework derived from cancer populations.

Different employment status, fertility status, fear of disease progression, social support, illness perception, patient global assessment of disease activity, and avoidance coping were collectively associated with PTG in patients with ADs, jointly explaining 49.3% of the total variance in PTG. This finding is consistent with the theoretical model of multifactorial interaction in PTG, suggesting that the occurrence and development of PTG are not driven by any single factor, but rather result from the interplay among sociodemographic characteristics, disease-related experiences, and psychosocial resources. Accordingly, clinical practice may consider adopting an integrated perspective to conduct comprehensive assessments and deliver individualized interventions for patients.

### 4.2 Positive factors and their positive associations with PTG in patients with ADs

#### 4.2.1 Social support as a core positive correlate of PTG

The results of this study showed that social support was significantly positively associated with PTG (β = 0.262, P < 0.001), which aligns with the consensus in chronic disease populations that “perceived social support is a core resource for psychological adaptation” (32). For patients with ADs characterized by a prolonged course and recurrent episodes, emotional care from family members, sustained companionship from friends, and professional guidance from healthcare providers are not only associated with emotional buffering but may also offer support at the levels of cognitive restructuring (e.g., constructing positive meaning of illness) and behavioral coping (e.g., promoting health management). These, in turn, are associated with patient growth in dimensions such as Relating to Others and Appreciation of Life. Furthermore, social support may be indirectly associated with the formation of PTG by alleviating patients’ negative illness perceptions and reducing avoidance coping behaviors, thereby serving as an important correlate linking disease-related stress and psychological growth.

In clinical practice, systematic attention may be given to activating patients’ social support networks. On one hand, family-based collaborative education models, treating patients and their caregivers as a coping dyad, may be considered for implementation. The digitally adapted version of the FOCUS (Family Oncology Caregiver Unmet Needs) evidence-based intervention program developed by Northouse et al. (33), as well as the couple-based intervention developed by Wang et al. (34) grounded in the “Live with Love” theoretical framework, both suggest that targeted dyadic interventions are associated with improvements in family members’ emotional support capacity and practical caregiving skills, and are also associated with mutual psychological growth from disease-related trauma for both partners. On the other hand, structured peer support networks may be considered for establishment. A peer mentoring intervention study conducted by Mosley-Williams et al. (35) among patients with SLE suggests that emotional resonance among patients and the transmission of practical disease management skills are associated with reduced avoidance coping and enhanced levels of activation in disease management, potentially reflecting the protective effects of social support.

#### 4.2.2 Moderate fear of disease progression and its positive association with PTG

In this study, fear of disease progression (FoP) was significantly positively associated with posttraumatic growth (PTG) (β = 0.233, P < 0.001). Previous research has suggested that the relationship between FoP and PTG may follow an inverted U-shaped curve, i.e., moderate disease-related fear is associated with the elicitation of PTG, whereas excessive fear may be associated with the inhibition of PTG (36). In the present sample, patients’ FoP levels were generally within a moderate range and had not reached the high-fear threshold that might be associated with PTG inhibition. Therefore, only a positive linear association between FoP and PTG was observed. It should be noted that, given the cross-sectional design of this study and the lack of direct testing for nonlinear relationships, the above discussion regarding the shape of the curve should be regarded as speculation based on previous research. Future studies may adopt longitudinal designs or nonlinear models to further examine this issue.

Autoimmune diseases are characterized by incurability and a relapsing course. In this context, moderate FoP is not purely pathological anxiety; instead, it may carry both adaptive vigilance-signaling value and significance for psychological adaptation. Such moderate fear is associated with behavioral tendencies including increased alertness, active seeking of disease-related knowledge, adherence to treatment recommendations, and mobilization of social resources. These tendencies may, in turn, be associated with growth in dimensions such as Personal Strength and Spiritual Change.

This finding has implications for clinical practice. Clinicians need not simply classify patients’ fear of disease progression as a negative emotion; rather, they may attempt to distinguish between adaptive fear and pathological anxiety. The goal of intervention may not necessarily be to eliminate fear, but possibly to guide and utilize moderate fear. For example, healthcare professionals may use techniques such as motivational interviewing and cognitive restructuring to help patients connect fear-related emotions to concrete, positive health management goals and behaviors. However, it should be noted that when fear of disease progression exceeds a moderate range, it may co-occur with pathological anxiety. Excessive negative emotions may deplete patients’ psychological regulatory resources and may be associated with inhibition of PTG. Previous studies have proposed assessment thresholds for fear of disease progression (FoP) in different disease populations: for breast cancer patients, a FoP score ≥ 36 is often used as the cut-off for high fear (36); for colorectal cancer patients, the corresponding threshold is 34 (37); while research on chronic non-malignant diseases suggests that a score range of 25–35 may represent the moderate fear interval within which FoP exerts its positive facilitative effects (38). To date, no specific threshold has been reported for the relationship between FoP and PTG in patients with autoimmune diseases. Future research may further explore the cut-off criteria specific to this population.

### 4.3 Negative factors and their associations with PTG in patients with ADs

#### 4.3.1 Factors negatively associated with PTG: negative illness perception, high symptom burden, and avoidance coping

Illness perception (β = –0.209, P < 0.001), self-assessed disease activity (β = –0.207, P < 0.001), and avoidance coping (β = –0.174, P = 0.001) were significantly negatively associated with PTG, suggesting that these factors may co-occur with lower levels of positive psychological adaptation in patients. Negative illness perception reflects patients’ pessimistic appraisal of disease severity and prognosis. This cognitive tendency is often associated with feelings of helplessness and hopelessness, which may make it more difficult for patients to reconstruct meaning from their illness experience. Higher self-assessed disease activity indicates that patients are experiencing pronounced physical discomfort, where pain experiences and psychological stress mutually reinforce one another and may be related to the depletion of psychological resources needed for cognitive restructuring. Meanwhile, although avoidance coping may be associated with short-term relief of emotional distress, over the long term it may be associated with fewer opportunities for patients to confront the trauma, process emotions, and integrate their illness experience, thereby showing a weaker association with the pathway from “trauma exposure” to “meaning construction” (40). These three factors may interrelate and collectively be associated with lower levels of PTG: negative cognitions are positively associated with symptom distress, symptom burden is positively associated with avoidance tendencies, and avoidance behaviors may be associated with reduced cognitive adjustment.

These findings suggest that, compared to objective clinical indicators, patients’ subjective perceptions of their illness, symptom experiences, and coping patterns may have a more direct relationship with their psychological growth. In clinical practice, systematic assessment of patients’ illness perceptions, symptom burden, and coping styles may be considered, with cognitive restructuring, symptom management, and adaptive coping training as areas of focus in psychological intervention. A web-based lifestyle intervention for patients with multiple sclerosis conducted by Bevens et al. (39) showed that through dietary optimization, exercise, and mindfulness training, patients experienced an enhanced sense of disease control and gradually achieved “symptom-cognition separation,” reducing automatic negative cognitions such as “symptom exacerbation equals disease deterioration.” This experience may have reference value for the autoimmune disease population. For patients with a tendency toward avoidance coping, graded exposure (targeting information avoidance) and narrative therapy (targeting emotional avoidance) may be attempted, combined with meaning-centered therapy, to assist patients in reinterpreting the value of their illness experience. Through systematic interventions, it may be possible to help patients gradually shift away from negative cognitions and avoidance patterns, thereby releasing psychological resources and creating conditions conducive to PTG.

#### 4.3.2 Employment and parenthood: associations with PTG and the possible mediating role of psychological burden

This study found that patients who were employed or had children exhibited significantly lower levels of PTG compared to those who were unemployed or childless. This difference may be related to the crowding-out effect of role conflict on psychological resources. Patients with autoimmune diseases need to engage in long-term disease management, investing substantial time and energy in maintaining their own health. Employed patients must cope simultaneously with work-related stress and disease treatment, while those with children need to balance disease management with parenting responsibilities. The conflicts in time and energy allocation arising from these dual responsibilities may be associated with reduced psychological resources for introspection and positive adjustment, which in turn may co-occur with lower levels of PTG.

In clinical practice, special attention may be given to patients with autoimmune diseases who bear heavier role burdens, particularly those who are employed or have children. To address the negative association between role conflict and PTG, interventions may consider focusing on psychological resource replenishment and health environment reshaping, rather than requiring patients to “strive harder for balance.” Drawing on the micro-mindfulness paradigm of van der Lee et al. (11), specific interventions could include developing 5–10 minute fragmented practice sessions to help patients rapidly restore emotional balance during commutes, work breaks, or after children have gone to sleep, which may help reduce psychological resource depletion. Concurrently, based on the e-health intervention experience of Ratanasiripong et al. (4), online support groups may be attempted for employed patients and those with parenting responsibilities, providing actionable cognitive templates rather than generalized reassurance.

### 4.4 Mediating pathways in the association between fear of disease progression and PTG

Path analysis revealed possible pathway patterns in the association between fear of disease progression (FoP) and posttraumatic growth (PTG). Within the scope of the present sample, FoP was not only directly positively associated with PTG, but was also indirectly associated with PTG through three parallel mediating pathways: social support, self-assessed disease activity, and avoidance coping.

First mediating pathway: FoP → social support → PTG (mediating effect accounted for 12.80%). In this study, FoP was positively associated with social support, and social support was positively associated with PTG. This suggests that a moderate level of fear of disease progression may co-occur with a tendency for patients to actively seek social support, and that a higher level of social support is associated with a higher level of PTG. This pathway can be summarized as a positive association pattern of “stress emotion → support seeking → psychological growth.”.

Second mediating pathway: FoP → patient global assessment of disease activity → PTG (mediating effect accounted for 11.37%). FoP was positively associated with self-assessed disease activity, and self-assessed disease activity was negatively associated with PTG. This suggests that fear of disease progression may co-occur with greater attention to one’s own disease activity, and that a negative self-assessment of disease activity is associated with a lower level of PTG. This pathway can be summarized as a negative association pattern of “stress emotion → negative experience → lower growth.”.

Third mediating pathway: FoP → avoidance coping → PTG (mediating effect accounted for 8.77%). FoP was positively associated with avoidance coping, and avoidance coping was negatively associated with PTG. This suggests that a higher level of fear of disease progression may co-occur with avoidance coping behaviors in some patients, and that avoidance coping behaviors are associated with a lower level of PTG. This is also a negative association pathway.

The three mediating pathways differ in direction (one positive, two negative). However, the total indirect effect was positive (0.132, 95% CI: 0.066–0.204), indicating that the association between FoP and PTG is not simply facilitative or inhibitory; rather, it may involve intertwined patterns of association through the activation of different psychosocial processes, with a net positive association. This may explain why FoP emerged as a positive predictor (based on statistical association) in the regression model, even though some of its mediating pathways pointed to intermediate variables that are negatively associated with PTG.

In summary, the association between FoP and PTG is complex. In the present sample, moderate FoP was directly positively associated with PTG, and may also be indirectly positively associated with PTG by prompting patients to seek social support. Higher levels of FoP, on the other hand, may co-occur with increased negative experience of disease activity and the elicitation of avoidance coping behaviors, thereby being indirectly negatively associated with lower levels of PTG. Based on the above association patterns, clinical interventions may consider multi-target and differentiated strategies. First, healthcare professionals may focus on assessing patients’ fear levels and communicating with them, using targeted psychological interventions to help maintain fear within a moderate range and to encourage its transformation into social support-seeking behaviors. Second, attempts may be made to interrupt the association pathways between FoP and catastrophic symptom perception or avoidance behaviors, using cognitive interventions to help reduce hypervigilance and negative interpretation of somatic sensations. The ultimate goal, without denying the adaptive value of fear, is to weaken its association pathways with psychological distress, thereby maximizing the net positive association between fear of disease progression and PTG.

### 4.5 Exploration of non-significant associative factors

In this study, the mediating effects of fear of disease progression (FoP) through resignation coping and illness perception did not reach statistical significance. Additionally, the direct effects of financial burden and number of comorbidities on PTG were also not statistically significant.

The non-significant mediating effect of FoP through resignation coping (indirect effect 4: β = 0.015, 95% CI: –0.004 to 0.051, P = 0.111) may be related to the high positive correlation between resignation coping and avoidance coping (r = 0.656, P < 0.01). The two share substantial variance, and avoidance coping had a stronger negative association with PTG (direct effect β = –0.174, P = 0.001; direct effect of resignation coping: β = –0.07, P = 0.233). In other words, there is multicollinearity between resignation coping and avoidance coping. Because avoidance coping had a stronger negative association with PTG, little unique variance was left for resignation coping to explain the relationship between FoP and PTG, making it difficult for its mediating pathway to reach statistical significance. This “suppression effect” resulting from multicollinearity may have masked the independent mediating role of resignation coping. Future studies may consider using larger sample sizes or factor merging techniques to further distinguish the independent contributions of the two variables.

The absence of significant direct effects for financial burden and number of comorbidities may be related to their possible indirect associations with PTG through mediating variables such as illness perception and social support. Furthermore, the multisystem involvement characteristic of autoimmune diseases may lead patients to subsume the stress of comorbidities within the overall burden of the primary disease, thereby attenuating the independent associative role of comorbidities. Subsequent studies could use structural equation modeling to further examine these indirect associative pathways.

## 5 Limitation

This study has preliminarily identified the key factors associated with PTG and their pathways in patients with ADs, although several limitations should be acknowledged. First, the sample was sourced from three hospitals in Anhui Province, China, using a convenience sampling method, which limits the representativeness of the sample; thus, the generalizability of the findings requires further validation. Second, all variables were measured using self-report scales. Although the Harman single-factor test indicated that common method bias was not severe, self-reports may still be influenced by recall bias, social desirability, and other factors, which may introduce some interference with the objectivity of the results. Third, this study employed a cross-sectional design, which precludes causal inferences about the relationships among variables or their dynamic changes. The mediating effects identified should be interpreted as estimates of the strength of associations between variables rather than as causal effects. Fourth, PTG is inherently a dynamic developmental process, and this study was unable to delineate the trajectory of changes in patients’ growth experiences. Future research may adopt longitudinal study designs to track its evolutionary patterns.

## 6 Conclusion

Based on the Transactional Model of Stress and Coping, this study constructed a structural equation model of factors associated with posttraumatic growth (PTG) in patients with autoimmune diseases. The results showed that PTG is synergistically associated with multiple factors, among which fear of disease progression emerged as a core correlate of PTG levels: this factor was not only directly positively associated with PTG, but was also indirectly associated with PTG through mediating pathways involving social support, self-assessed disease activity, and avoidance coping. These findings suggest that in clinical practice, attention may be paid to patients’ level of fear of disease progression, strengthening of social support provision, and reduction of avoidance coping behaviors, thereby creating favorable conditions for cultivating PTG in patients.

## 7 Future directions

Future research may further expand the sample size and adopt a multicenter, prospective cohort study design to explore the dynamic trajectories of posttraumatic growth in patients with autoimmune diseases and the long-term associative patterns between various influencing factors and posttraumatic growth. In addition, integrating qualitative research methods would enable a more in-depth depiction of the nuanced processes and turning points in patients’ growth experiences, providing a richer foundation for the development of culturally adapted and disease-specific psychological intervention programs.

## Data Availability

The original contributions presented in the study are included in the article/supplementary material. Further inquiries can be directed to the corresponding author.
